# Effects of unstable footwear on joint reactions and muscle forces: an inverse dynamics study

**DOI:** 10.1186/1757-1146-5-S1-O7

**Published:** 2012-04-10

**Authors:** Michael Schwarze, Frank Seehaus, Christof Hurschler, Hazibullah Waizy

**Affiliations:** 1 Laboratory for Biomechanics and Biomaterials, Hannover Medical School, 30625 Hannover, Germany; 2 Department of Orthopaedics, Hannover Medical School, 30625 Hannover, Germany

## Background

Unstable shoe designs should support the muscle activity and promise the treatment of leg, back and foot problems. According to manufacturers, they should activate additional muscles and reduce joint reaction forces. Goal of this study is to investigate the effect of an unstable shoe design to gait patterns of healthy volunteers and by the means of inverse dynamic multi-body simulation.

## Materials and methods

Seven subjects (age: 46.5±7.6 years, weight: 91.7±11.1kg) familiar with unstable shoes performed five trials of level walking in three testing conditions (barefoot, conventional and unstable shoe). As an unstable shoe the Anti-Step (Chung-Shi) was chosen. Kinematic and kinetic data was acquired with a motion capturing system (Vicon) and two forceplates (AMTI). The inverse dynamics model of the lower extremity consists of nine rigid bodies which are connected with idealized joints and a set of all relevant muscles.

## Results

**Figure 1:**
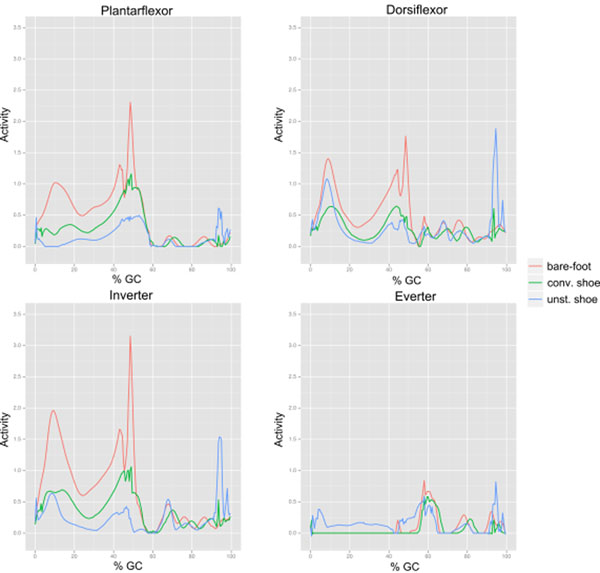
Muscle activation of the ankle muscle groups around the ankle for one subject. Lines representing results for barefoot (red), conventional shoe design (green) and unstable shoe design (blue).

**Figure 2:**
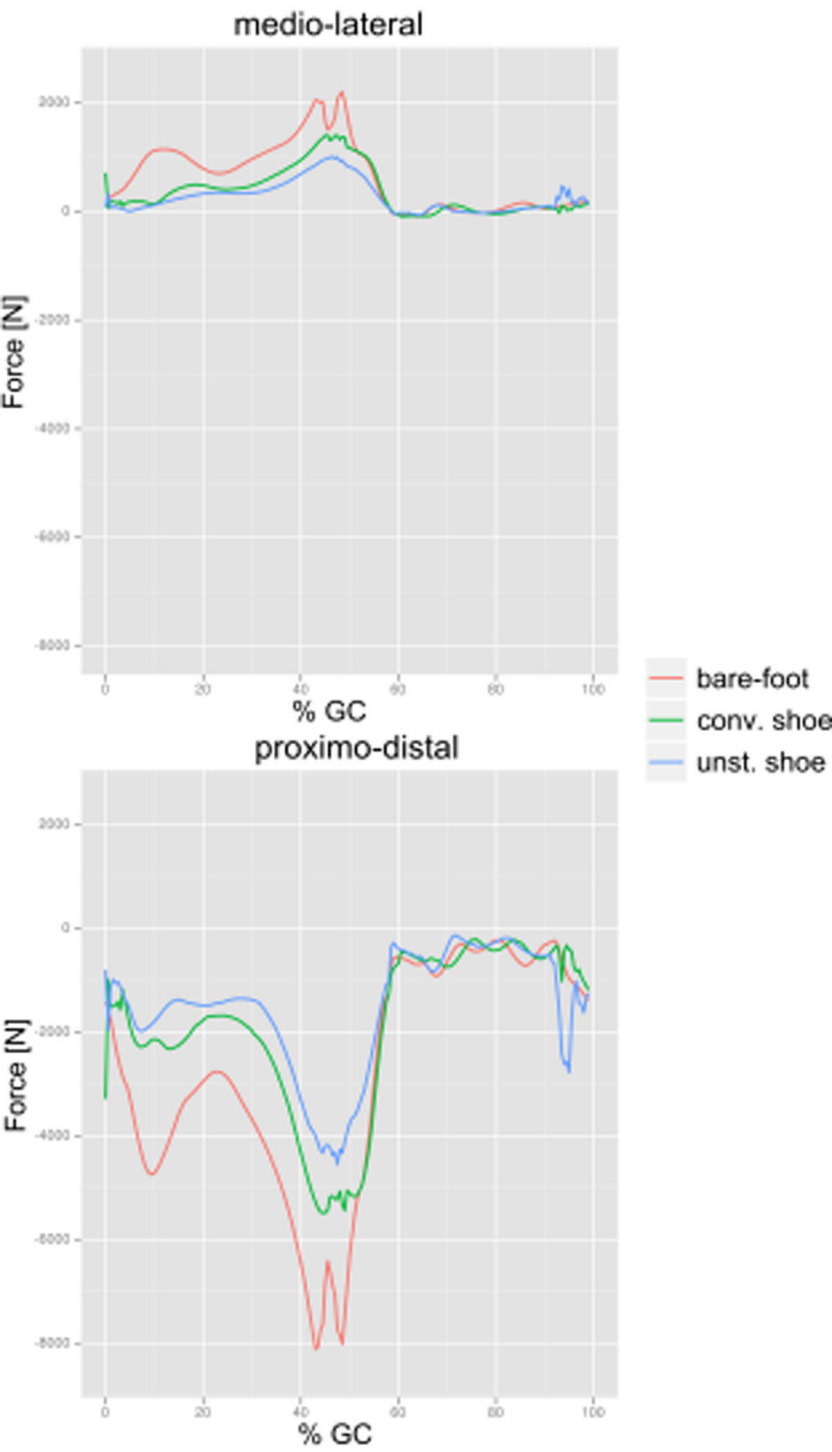
Joint reaction forces in the ankle for one subject. Lines representing results for barefoot (red), conventional shoe design (green) and unstable shoe design (blue).

Comparing walking speed while walking barefoot or with stable and unstable shoe designs, the volunteers walked significantly slower in the barefoot case (p<.003). Preliminary multi body-simulation data was analysed for four out of seven volunteers. Peak joint reaction forces were reduced by 29% when comparing conventional with unstable shoes (Figure 2). Muscle activation changes in magnitude for all groups (Figure 1). The timing remains similar, except the everter group activating only with the unstable shoe during stance phase.

## Conclusions

The simulation reveals muscle activation patterns that indicate instability along the inversion/eversion axis of the ankle, which is also found in the literature [1]. The additional activation of the everter group during stance phase possibly exercises this group and could lead to an effect on the arch of the foot.
